# Socioeconomic Factors Affecting Visual Acuity in Cataract Camps

**DOI:** 10.21203/rs.3.rs-8437303/v1

**Published:** 2026-01-16

**Authors:** Eunyoo Kim, Elizabeth White, Dongseok Choi, Dong-Wouk Park

**Affiliations:** Oregon Health & Science University

**Keywords:** Cataracts, world blindness, socioeconomic disparity, global health, cataract camp, blindness prevention

## Abstract

**Background:**

Cataracts are the leading cause of reversible blindness worldwide. Though temporary and resource-intensive, cataract camps provide large-scale treatment of the condition. This study sought to determine socioeconomic factors that affect short-term improvements in visual acuity observed in cataract camps across 17 countries.

**Methods:**

This observational study represents 52 camps in 17 low- and middle-income countries. Mixed-effects linear regression models were used to analyze associations between changes in LogMAR score of uncorrected visual acuity (UCVA) one to four days post-operation and socioeconomic factors including per capita GDP, Human Development Index, Cataract Surgical Rate, and density of in-country ophthalmologists.

**Findings:**

Among 4110 patients, after accounting for age and sex, LogMAR UCVA significantly decreased over the first four days postoperatively (p < 0·0001). This decrease was also associated with the number of ophthalmologists per million performing cataract surgery (p = 0·0227). Patients who underwent extracapsular cataract extraction (ECCE) exhibited a greater decrease in LogMAR UCVA per day post-op (p < 0·0001). Per capita GDP was not significantly associated with the change in LogMAR UCVA per day post-op.

**Interpretation:**

Our analyses suggest that cataract camps in areas with fewer ophthalmologists performing cataract surgery and ECCE surgeries correlated with the most improvement in vision. Targeting such populations and training local ophthalmologists to perform ECCE surgery may yield a higher impact in low-resource settings with a high burden of cataract-induced blindness.

## Introduction

Cataracts are the leading cause of avoidable blindness, accounting for nearly half of world blindness in 2020 [1]. There is a significant disparity in the prevalence of cataracts and access to treatment in socioeconomically vulnerable populations. This treatment gap is widening due to globally aging demographics [1-5]. Although the benifits of surgery have been well documented, there is scarce evidence on which strategies work best to address this inequity in low-resource settings [6].

A mobile eye surgical unit, or a “cataract camp,” has been suggested as a safe, viable, and cost-efficient model to address the backlog of cataract-induced blindness [7-10]. By bringing experienced foreign or local ophthalmologists to areas inaccessible to care, cataract camps could improve vision, quality of life, and potentially the local economy as well [9-11].

Although there have been numerous studies on the efficacy of cataract camps in one or a few countries, a multinational study that reports a larger and more diverse body of cataract camp data has been absent, making it challenging to apply findings from one country to another. Therefore, this study presents visual acuity data collected across 17 low- and middle-income countries (LMICs) by Vision Care, an international blindness relief organization that has operated over 300 short-term cataract camps since 2002.

The goal of the study is threefold. First, to assess the correlation of the visual acuity and known country-level socioeconomic factors including Gross Domestic Product (GDP), Human Development Index (HDI), and Cataract Surgical Rate (CSR). Second, to assess its correlation to existing ophthalmologist workforce in each country. Third, to assess the differences in cataract surgery outcomes based on the type of surgical technique utilized.

## Methods

This study was conducted in accordance with the tenets of the Declaration of Helsinki and was exempt from the institutional review board at Oregon Health and Science University.

### Study Population

This observational study utilized de-identified data from Vision Care. The patients underwent cataract surgery between 2013 and 2023. The information used for analysis included the date of surgery, age, sex, type of surgical technique, preoperative visual acuity, postoperative visual acuity, and the country in which the surgery was performed. Uncorrected visual acuities (UCVA) were measured on ordinal scales such as no light perception (LP−), light perception (LP+), count fingers (CF), or hand motion (HM) at varying distances, or on Snellen scales on Snellen chart, a number chart, or a tumbling E chart.

Only cataract extraction with intraocular lens (IOL) implant cases were included in the analysis. Cases with additional surgical treatments were excluded. For example, pterygium surgery, IOL exchange, IOL repositioning that may or may not have been paired with cataract extraction were excluded.

### Cataract Camp Settings

The cataract camps were carried out in areas where Vision Care was invited by the local government. This study reflects cataract camps in the following countries: Bangladesh, Cambodia, China, Kyrgyzstan, Mauritania, Mongolia, Morocco, Mozambique, Nigeria, Pakistan, Peru, Sri Lanka, Tanzania, Uganda, Uzbekistan, Vanuatu, and Vietnam. In each camp, locally available equipment was used, and unavailable equipment was brought in via air transportation.

Camp participants were trained volunteers or Vision Care staff, with each camp having a unique set of members with a few returning volunteers. Surgical type was determined by the operating surgeon following a slit lamp exam. Individual IOL calculation and intraocular pressure (IOP) measurement were performed preoperatively. In general, extracapsular cataract extraction (ECCE) in a manual small incision cataract surgery (MSICS) fashion was preferred for dense and mature cataracts. Phacoemulsification was preferred if the cataract was deemed soft. The operating ophthalmologists were trained in the United States or South Korea, with a few local ophthalmologists operating under direct supervision of experienced surgeons. Operated patients were followed up the morning after the surgery and again on the last day of the camp for a second evaluation. During the one-week camp period, surgeries were performed Monday through Thursday, and Friday was dedicated to emergency cases and final follow-ups.

### Socioeconomic Factors

This study used country GDP per capita in 2021 constant international dollars with the purchasing power parity (PPP) calculations to adjust for economic inflation and account for differences in cost of living [12]. The HDI summarizes the following four dimensions of socioeconomic factors: life expectancy, mean years of schooling, expected years of schooling, and gross national income (GNI) per capita [13]. The CSR reports the number of cataract surgeries performed per million populations in a year [14]. The number of ophthalmologists and the number of ophthalmologists performing cataract surgery per capita by country were obtained from a study published in 2020 by Resnikoff and colleagues [15].

### Statistical Analysis

The visual acuity data were converted into a LogMAR scale for ease of comparison. Snellen values were converted using the formula below:

LogMAR UCVA=−1∗log10(Fraction as a Decimal)

Visual acuities measured by various distances of hand motion (HM) and count finger (CF) were converted to LogMAR UCVA using methods previously described,using the mean distal interphalangeal (DIP) joint width, including inter-digit spaces, of 86.07mm [16-18].

To assess for changes in LogMAR UCVA between pre-operation and one, two, three, or four days post-operation, four paired Wilcoxon tests were conducted. Bonferroni corrections were applied to p-values.

Multivariable mixed-effects linear regression models were used to assess for the change in LogMAR UCVA over the first four days after surgery, accounting for age and sex, both overall as well as within each country. Similar models were used to assess for interactions between the change in LogMAR UCVA over the first four days after surgery, accounting for patient age and sex, and the following variables: surgery type, GDP PPP per capita, HDI, CSR, number of ophthalmologists per capita, and number of ophthalmologists per capita performing cataract surgery. After accounting for GDP PPP, the interactions between number of ophthalmologists, number of ophthalmologists performing cataract surgery, and HDI were also assessed.

## Results

Data was collected on 4120 eyes from 4110 patients from 52 cataract camps held between February 2013 and November 2023. Ages ranged from 3 to 109 years, with a mean of 66·9 years (Table 1). 55·2% of patients were female. 78·9% of patients underwent phacoemulsification, 20·6% underwent ECCE, and 0·4% underwent intracapsular cataract extraction (ICCE). Cataract operations were performed in 17 countries: Bangladesh, Cambodia, China, Kyrgyzstan, Mauritania, Mongolia, Morocco, Mozambique, Nigeria, Pakistan, Peru, Sri Lanka, Tanzania, Uganda, Uzbekistan, Vanuatu, and Vietnam. Most data were reported from China (15·9%), Morocco (15·6%), Tanzania (8·1%), Kyrgyzstan (8%), or Uzbekistan (7·7%). Only 1·2% of data were from Sri Lanka.

### Pre-Operative Visual Acuity

Preoperative visual acuities were available for 3976 surgeries from 3955 eyes from 3945 patients. Among the 3976 surgeries with documented preoperative visual acuities, 3060 (77%) presented with a visual acuity of 20/200 or worse. As compared to ECCE, phacoemulsification eyes had significantly lower preoperative logMAR UCVA by an average of 0·834 units (p < 0·0001).

There was a significant association between preoperative logMAR UCVA and GDP PPP per capita (p < 0·0001), with preoperative logMAR UCVA decreasing by an average of 0·028 units for every 1,000-unit increase in GDP PPP per capita (95% CI: 0·022 to 0·033). There was a significant association between preoperative logMAR UCVA and HDI (p < 0·0001), with preoperative logMAR UCVA decreasing by an average of 0·9 units for every 1-unit increase in HDI (95% CI: 0·62 to 1·18). There was a significant association between preoperative logMAR UCVA and CSR per million (p = 0·0002), with preoperative logMAR UCVA decreasing by an average of 0·073 units for every 1,000-unit increase in CSR per million (95% CI: 0·035 to 0·11). There was a significant association between preoperative logMAR UCVA and the number of ophthalmologists per million citizens performing cataract surgery (p < 0·0001), with preoperative logMAR UCVA decreasing by an average of 0·053 units for every 1 additional ophthalmologist per million citizens performing cataract surgery (95% CI: 0·045 to 0·061).

There was not a significant association between preoperative logMAR UCVA and the number of ophthalmologists per million citizens (p = 0·2741).

### Changes in Visual Acuity

The mean LogMAR UCVA for all patients changed from 1·66 preoperatively to 0·964 one day postoperatively, 0·864 at two days, 0·805 at three days, and 0·79 at four days. There was a significant improvement from preoperative visual acuity at one day (p < 0·0001), two days (p < 0·0001), three days (p < 0·0001), and four days (p < 0·0001) postoperatively. (Table 2).

There was a significant association between calculated LogMAR UCVA and days post-operation (p < 0·0001; conditional R^2^ = 0.42), with LogMAR UCVA decreasing by an average of 0·286 units per day postoperation (95% CI: 0·274 to 0·298; Figure A1). This significant decrease in LogMAR UCVA per day was consistent across all 17 countries (p < 0·001).

After accounting for age and sex, there was a significant interaction between the change in logMAR UCVA per day after surgery and preoperative logMAR UCVA (p < 0·0001), with logMAR UCVA decreasing by an additional 0·054 units for every 1 additional unit in preoperative logMAR UCVA (95% CI: 0·041 to 0·067 additional units).

**Figure 1:**
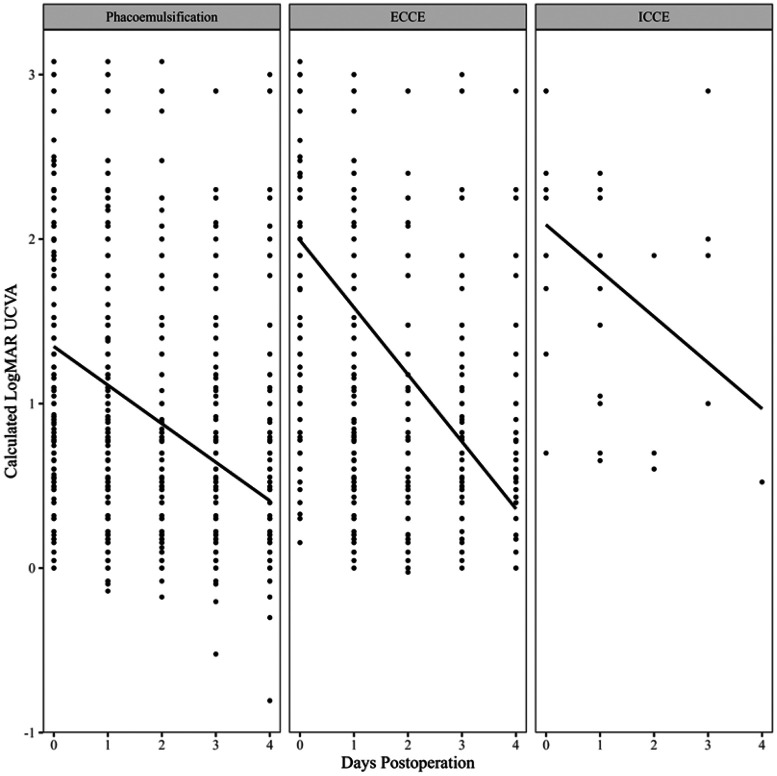
Change in LogMAR UCVA per day postoperation by type of surgical technique.

As compared to patients who underwent phacoemulsification, patients who underwent ECCE exhibited a greater decrease in LogMAR UCVA per day (p < 0·0001), decreasing by 0·175 additional units per day (95% CI: 0·146 to 0·205; Figure 1).

The decrease in LogMAR UCVA per day was significantly greater in countries with fewer ophthalmologists per million performing cataract surgery (p < 0·0001). Between the first quartile of number of ophthalmologists performing cataract surgery per million citizens of 1·7 and the third quartile of 9·9, the average decrease in LogMAR UCVA per day post-operation decreased from 0·352 to 0·274 units per day. This association remained after accounting for GDP PPP per capita (p = 0·0024).

The decrease in LogMAR UCVA per day was not significantly associated with the ophthalmologists per million (p = 0·7462), HDI (p = 0·716), CSR per million (p = 0·7532), or GDP PPP per capita (p = 0·8059).

## Discussion

In this study, we reported the outcomes of 52 cataract camps from 17 LMICs. Our global data demonstrates a highly significant and consistent improvement in visual acuity from day one through four (p < 0·0001) after the surgery. This trend was universal across all countries in this study, suggesting a strong argument for the model’s replicability and generalizability.

We found that the rate of improvement in vision was significantly higher in patients who presented with worse preoperative vision. This tells us that cataract surgery brings the most benifits to the population with the highest burden of unoperated, longstanding cataracts, which contribute to avoidable blindness in many LMICs. The rate of improvement was also significantly higher in those who underwent ECCE surgery than phacoemulsification. The observed faster initial recovery with the ECCE/MSICS method supports its use as a pragmatic approach in low-resource settings for treating severe cataracts, consistent with previous studies [9, 19].

Contrary to expectations, broader national-level indicators, including total ophthalmologists per capita, HDI, CSR, and GDP PPP, were not significantly associated with the daily rate of visual recovery. (p > 0·7 for all) This suggests that these short-term postoperative outcomes are more strongly influenced by individual-level factors, such as baseline UCVA and surgical type, rather than by macroeconomic context. This finding appears to contrast with prior reports on global disparities in visual impairment, which emphasized the importance of economic indicators in estimating healthcare access and visual outcomes [2-4]. One possible explanation lies in the site selection process of Vision Care. The camp sites are typically chosen based on requests from local governments or partner organizations, selecting areas with higher demand for cataract surgery. As a result, the national socioeconomic indices used in this analysis may not accurately reflect the specific regions where Vision Care operates, particularly considering the substantial disparities in wealth and healthcare access within each country.

Notably, however, the daily rate of visual improvement was significantly higher in countries with the lowest density of local cataract surgeons (p < 0·0001), a relationship that remained significant even after controlling for national GDP PPP (p = 0·0024). This suggests that cataract camps are most impactful where local eye care infrastructure is the weakest, further supporting the role of cataract camp as an effective and essential mechanism for closing the growing treatment gap in socioeconomically vulnerable regions. The persistence of this association after controlling for GDP PPP emphasizes that the scarcity of trained surgeons, rather than general economic development, is the primary constraint that cataract camps are designed to overcome. This result is particularly relevant in the context of globally aging population and the subsequently increasing demand for eye health services including cataract surgery.

Given these results, our study strongly advocates the importance of training and incentivizing local ophthalmologists in LMICs to perform cataract surgery, with or without the aid of cataract camps. Organizations like Vision Care have been actively involved in equipping local eye care providers and ophthalmologists to expand vision screening and perform cataract surgeries. These educational initiatives can not only improve the efficiency of cataract camps but also strengthen local ophthalmologists by offering direct hands-on training from experienced surgeons. Given the marked shortage of ophthalmologists in LMICs, training competent eye care professionals who take on the challenge locally will be critical in achieving a sustainable eye care model [4, 20].

This study has several limitations inherent to the analysis of observational data from a mass campaign format. First, our visual acuity data is limited to uncorrected visual acuity (UCVA) and does not include best-corrected visual acuity (BCVA). While UCVA is a practical and relevant outcome for evaluating the functional success of cataract camps, it may not capture the full rehabilitative potential. Second, the follow-up period is limited to the duration of the camp, preventing an assessment of long-term surgical outcomes, stability of vision, or post-operative complication rates. Yet, previous studies have suggested that such short-term visual acuity is still a valuable quality control measure in areas with poor follow-up rates [21, 22]. Finally, the use of macro-level socioeconomic indicators may obscure heterogeneity within each country.

To our knowledge, this study is the largest studies of multinational cataract camps, involving data from 17 LMICs and over four thousand patients. We conclude that cataract camps may yield the greatest impact in populations with the most severe cataracts and in countries facing the greatest shortage of local cataract surgeons. To tackle the growing global challenge of vision impairment, clinicians and policymakers must continue to support high-volume, targeted interventions like cataract camps while simultaneously focusing on building sustainable local surgical capacity.

## Supplementary Material

This is a list of supplementary files associated with this preprint. Click to download.

Appendix.docx

## Figures and Tables

**Table 1:** Patient demographics.

	N = 4,110^1^
**Age (years)**	66·9 (3·0, 109·0)
*(Missing)*	5
**Sex**	
Female	2,269 (55%)
Male	1,840 (45%)
*(Missing)*	1
**Operation Type**	
ECCE	847 (21%)
ICCE	15 (0·4%)
Phacoemulsification	3,243 (79%)
*(Missing)*	5
**Country**	
Bangladesh	186 (4·5%)
Cambodia	97 (2·4%)
China	654 (16%)
Kyrgyzstan	330 (8·0%)
Mauritania	180 (4·4%)
Mongolia	68 (1·7%)
Morocco	643 (16%)
Mozambique	219 (5·3%)
Nigeria	101 (2·5%)
Pakistan	300 (7·3%)
Peru	56 (1·4%)
Sri Lanka	49 (1·2%)
Tanzania	332 (8·1%)
Uganda	232 (5·6%)
Uzbekistan	317 (7·7%)
Vanuatu	53 (1·3%)
Vietnam	293 (7.1%)

1 Mean (Min, Max); n (%)

**Table 2:** Summary of LogMAR UCVA preoperation and one, two, three, or four days postoperation.

	Preoperation N = 3,955^1^	One Day Postoperation N = 3,591^1^	Two Days Postoperation N = 798^1^	Three Days Postoperation N = 741^1^	Four Days Postoperation N = 483^1^
**LogMAR UCVA**	1·657 (0·000, 3·079)	0·962 (−0·140, 3·079)	0·865 (−0·176, 3·079)	0·807 (−0·523, 3·000)	0·789 (−0·806, 3·000)
**Median decrease in LogMAR UCVA**	··	0·650	0·875	0·835	0·778
**P-Value**	··	< 0·0001	< 0·0001	< 0·0001	< 0·0001

1 Mean (Min, Max)
